# “Inflamm‐aging” influences immune cell survival factors in human bone marrow

**DOI:** 10.1002/eji.201646570

**Published:** 2017-01-11

**Authors:** Luca Pangrazzi, Andreas Meryk, Erin Naismith, Rafal Koziel, Julian Lair, Martin Krismer, Klemens Trieb, Beatrix Grubeck‐Loebenstein

**Affiliations:** ^1^ Department of ImmunologyInstitute for Biomedical Aging ResearchUniversity of InnsbruckInnsbruckAustria; ^2^ Department of Molecular and Cell BiologyInstitute for Biomedical Aging ResearchUniversität InnsbruckInnsbruckAustria; ^3^ Department of Orthopedic SurgeryInnsbruck Medical UniversityInnsbruckAustria; ^4^ Department of Orthopedic SurgeryHospital Wels‐GrieskirchenWelsAustria

**Keywords:** Aging, Bone marrow, Immunosenescence, Immunological memory, ROS

## Abstract

The bone marrow (BM) plays a key role in the long‐term maintenance of immunological memory. However, the impact of aging on the production of survival factors for effector/memory T cells and plasma cells in the human BM has not been studied. We now show that the expression of molecules involved in the maintenance of immunological memory in the human BM changes with age. While IL‐15, which protects potentially harmful CD8^+^CD28^−^ senescent T cells, increases, IL‐7 decreases. IL‐6, which may synergize with IL‐15, is also overexpressed. In contrast, a proliferation‐inducing ligand, a plasma cell survival factor, is reduced. IFN‐y, TNF, and ROS accumulate in the BM in old age. IL‐15 and IL‐6 expression are stimulated by IFN‐y and correlate with ROS levels in BM mononuclear cells. Both cytokines are reduced by incubation with the ROS scavengers N‐acetylcysteine and vitamin C. IL‐15 and IL‐6 are also overexpressed in the BM of superoxide dismutase 1 knockout mice compared to their WT counterparts. In summary, our results demonstrate the role of inflammation and oxidative stress in age‐related changes of immune cell survival factors in the BM, suggesting that antioxidants may be beneficial in counteracting immunosenescence by improving immunological memory in old age.

## Introduction

One of the most prominent changes of the aging immune system is the involution of the thymus, which is responsible for a dramatic early decline in the generation of new naïve T cells 1, 2. For this reason, adaptive immunity in old age is mainly supported by a turnover of an existing population of antigen‐experienced cells, the maintenance of which is crucial to fight infections 3. Recent studies performed in both mice and humans describe the BM as a preferred site for the survival of effector/memory CD4^+^ and CD8^+^ T cells and long‐lived plasma cells 4, 5, 6, 7, 8, 9, 10. Survival factors promote long‐term maintenance of effector/memory cell subpopulations. Studies in mice suggest that long‐lived plasma cells reside in survival niches organized by reticular stromal cells expressing the chemokine CXCL‐12 (stromal cell‐derived factor 1; SDF1) 11. Survival factors such as a proliferation‐inducing ligand (APRIL) and IL‐6, mainly produced by myeloid cell types, are also known to be important for the maintenance of long‐lived plasma cells 12, 13, 14, 15, 16. Effector/memory CD4^+^ T cells have been described also to migrate to BM niches where they interact with VCAM‐1^+^ stromal cells producing the survival factor IL‐7 5, 17, 18. Maintenance of effector/memory CD8^+^ T cells also requires IL‐7 19. The cytokine IL‐15 can contribute to this process, and it is particularly important for the preservation of highly differentiated CD8^+^ effector T cells 20, 21, 22, 23. DCs, monocytes (CD14^+^), CD34^+^ hematopoietic progenitor cells and BM stromal cells have been described to produce IL‐15 24, 25. In particular, IL‐15 transpresented by DCs and CD14^+^ cells is important for effector/memory CD8^+^ T‐cell generation and maintenance 26, 27, 28.

One hallmark of immunosenescence is the accumulation of highly differentiated effector CD8^+^ T cells, which lack the expression of the costimulatory molecule CD28 29, 30, 31. It has been suggested that high numbers of CD8^+^CD28^−^ T cells are detrimental for elderly people, as their accumulation is associated with high inflammation levels and an increased risk of age‐related diseases and mortality 32, 33. Since IL‐15 and IL‐6 have been shown to contribute to the generation and maintenance of CD8^+^CD28^−^ T cells, the expression of these cytokines should be controlled in order to guarantee sufficient BM space for CD4^+^ helper cells and plasma cells in old age, by avoiding the accumulation of exhausted T cells 34, 35.

In the present study, we analyzed the expression of effector/memory cell survival factors in the BM of people of different ages, showing that the levels of many of these molecules change with age. IL‐15 and IL‐6 could be stimulated with IFN‐γ and correlate with ROS levels in the BM mononuclear cell (BMMC) population. The expression of both cytokines could be reduced by incubation of the cells with the ROS scavengers N‐acetylcysteine (NAC) and vitamin C. The importance of ROS for the regulation of BM IL‐15 and IL‐6 production and the accumulation of CD8^+^ effector cells was further confirmed by results from a mouse model. This suggests that inflammation and oxidative stress may lead to age‐related impairments in the maintenance of immunological memory in old age by changing the expression pattern of effector/memory cell survival factors in the BM. and by promoting the persistence of highly differentiated CD8^+^ CD28^−^ T cells in the BM as a consequence.

## Results

### Effector/memory cell survival factors are differentially expressed in BMMCs during aging

**Figure 1:**
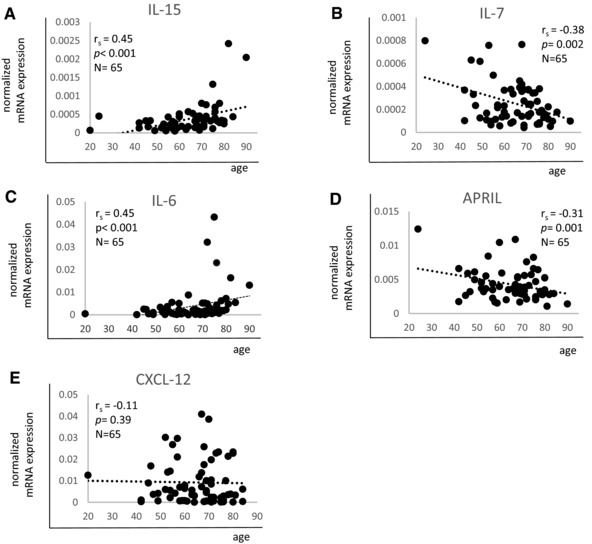
mRNA expression of effector/memory cell survival factors in BMMCs from persons of different ages. Expression levels of (A) IL‐15, (B) IL‐7, (C) IL‐6, (D) APRIL, and (E) CXCL‐12 in correlation with age are shown. mRNA expression of each gene was measured by qRT‐PCR and normalized against the housekeeping gene β‐actin. Spearman coefficient (*r*_s_), *p* value and sample size (*N*) are shown in each graph. Data were pooled from five independent representative experiments with 13 samples each. A total of 65 samples were included in the analysis.

**Figure 2:**
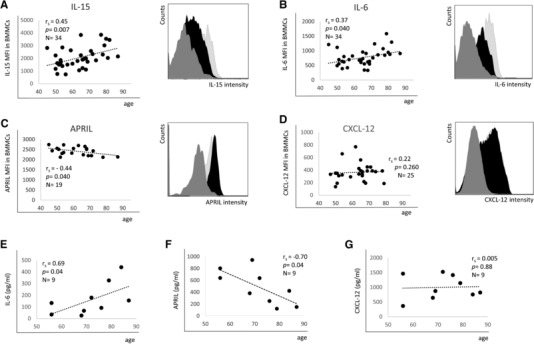
Protein expression of effector/memory cell survival factors in BMMCs from persons of different ages. The MFI of (A) IL‐15, (B) IL‐6, (C) APRIL, and (D) CXCL‐12 measured by FACS analysis in correlation with age is shown. For each survival factor a representative FACS histogram demonstrating cells from one young (black) and one old (light gray) donor as well as the isotype control (dark gray) is shown. Data were pooled from 15 independent representative experiments with 1–3 samples each. (E) IL‐6, (F) APRIL, and (G) CXCL‐12 expression measured in supernatants from BMMCs cultured for 5 h. Results are shown in correlation with age. Spearman coefficient (*r*_s_), *p* value and sample size (*N*) are shown in each graph. Data were collected in one representative experiment with nine samples.

Whether aging influences the levels of molecules important for the maintenance of immunological memory in the BM is unknown. We first analyzed the expression of effector/memory cell survival factors at the mRNA level in BMMCs from 65 donors with an age range of 20–90 years (Fig. 1). We found that IL‐15 and IL‐6 mRNA increase with age, whereas the expression of IL‐7 and APRIL decrease (Fig. 1A–D). IL‐15 and IL‐6 levels were very low and in some samples almost undetectable in persons under 65 years, while they were frequently higher in the age group >65 years. Interestingly, some older donors had a very high expression of both cytokine's mRNAs. IL‐15 mRNA was 2.1 ± 0.1‐fold (*p* = 0.01) and IL‐6 mRNA 3.2 ± 0.2‐fold (*p* = 0.04) higher in persons over 65 years compared to the donors below 65 years. The mRNA expression of IL‐7 and APRIL was heterogeneous in younger donors while it was uniformly low in the oldest donor group. IL‐7 levels were 1.9 ± 0.1‐fold (*p* = 0.01) and APRIL 1.5 ± 0.1 fold lower in persons >75 years compared to the age group of <75 years. No age‐related changes were observed for the chemokine CXCL‐12, with great deviations at all ages (Fig. 1E). Flow cytometry experiments confirmed the results described for mRNA at the protein level (Fig. 2). IL‐15 and IL‐6 levels increased while APRIL decreased and CXCL‐12 did not change with age. Age‐related changes were small, but the correlations between survival factors and age were highly significant. The age‐related changes of IL‐6 and APRIL were confirmed by measuring the secreted cytokines using ELISA (Fig. 2E–F). The secretion of CXCL‐12 did not change in old compared to younger donors (Fig. 2G). IL‐7 immunofluorescence could not be performed since no flow cytometry antibody (Ab) is available for this cytokine. Neither IL‐15 nor IL‐7 could be detected in the supernatants by ELISA, presumably due to concentrations under the detection level of the assays. The results indicate that the expression of molecules important for the long‐term maintenance of effector/memory T cells and long‐lived plasma cells in the BM changes during aging.

### Aging affects expression of effector/memory cell survival factors in subpopulations of BMMCs

**Figure 3:**
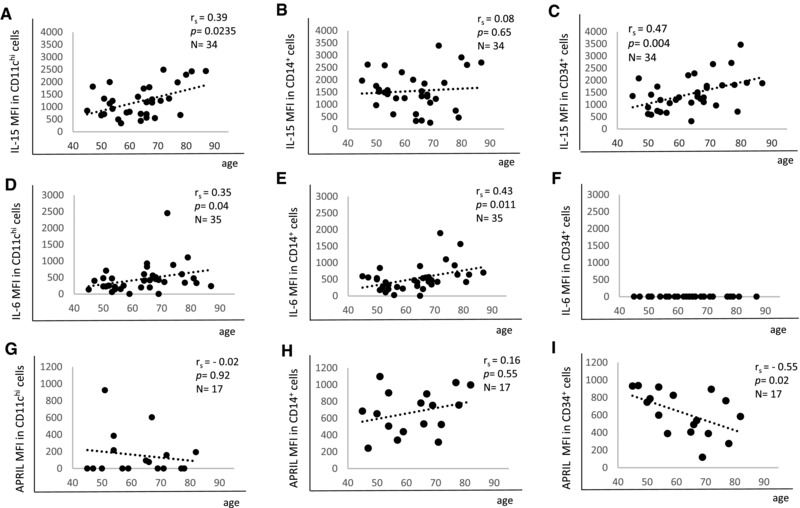
Protein expression of effector/memory cell survival factors. BMMC subpopulations from persons of different ages were analyzed by intracellular immunofluorescence staining followed by FACS analysis. The MFI of (A–C) IL‐15, (D–F) IL‐6, and (G–I) APRIL in CD11c^hi^, CD14^+^, and CD34^+^ cells in correlation with age is shown. Spearman coefficient (*r*_s_), *p* value and sample size (*N*) are shown in each graph. Data were pooled from 15 independent experiments with 1–3 samples each.

Antigen‐presenting CD11c^hi^ and CD14^+^ cells have been considered to be the main cell types producing APRIL and IL‐6 and producing and trans‐presenting IL‐15 to T cells in the BM 14, 16, 25. CD34^+^ myeloid progenitor cells have also been found to produce IL‐15 24. Here, we investigated which of these BM cell subpopulations were responsible for the age‐related changes of effector/memory cell survival factors observed in BMMCs. The respective gating strategies are shown in Supporting Information Fig. 1A. The size of the three subpopulations did not change with age (Supporting Information Fig. 1B–D). IL‐15 levels increased in old age in CD11c^hi^ and CD34^+^ cells, but did not change in CD14^+^ cells (Fig. 3A–C). A similar staining intensity for IL‐15 was detected in all three subpopulations. IL‐6 was upregulated with age in both CD11c^hi^ and CD14^+^ cells (Fig. 3D, E) but was not expressed by CD34^+^ cells (Fig. 3F). Again, staining intensity was similar in CD11c^hi^ and CD14^+^ cells. In contrast, APRIL expression negatively correlated with age in CD34^+^ cells while there was no correlation with age in CD11c^hi^ and CD14^+^ cells (Fig. 3G–I). In most donors, APRIL was not expressed in CD11c^hi^ cells, while similar expression levels were found in CD14^+^ and CD34^+^ cells among the different donors. The dot plots for every cytokine in one younger and one old donor in the three subpopulations is shown in Supporting Information Fig. 2.

### Accumulation of proinflammatory molecules and ROS in the aged BM

**Figure 4:**
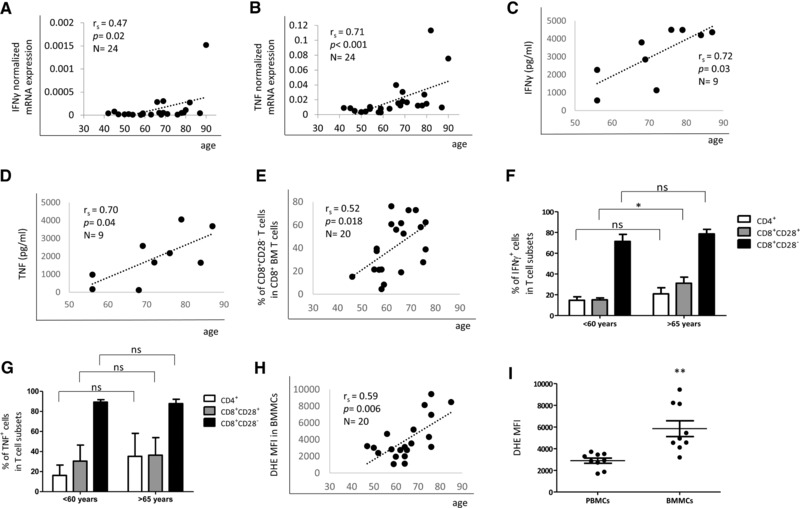
IFN‐y and TNF production as well as ROS levels increase in the BM in old age. mRNA expression of (A) IFN‐γ and (B) TNF in correlation with age is shown. The mRNA expression of each gene was measured by qRT‐PCR and normalized against the housekeeping gene β‐actin. Data were collected in one representative experiment with 24 samples. IFN‐γ (C) and TNF (D) expression measured by ELISA in supernatants from BMMCs stimulated with PMA and ionomycin in correlation with age. Data were collected in one experiment performed with nine samples. (E) Percentages of CD8^+^ CD28^−^ T cells within CD8^+^ cells in correlation with age. Spearman coefficient (*r*_s_), *p* value and sample size (*N*) are shown in each graph. Data were collected in ten experiment with two samples each. Percentages of IFN‐γ‐ (F) and TNF‐ (G) producing CD4^+^, CD8^+^CD28^+^, and CD8^+^CD28^−^ T cells from BMMCs in donors < 60 years (mean 54 ± 4.8 years, age range 46–59) and >65 years (mean 72 ± 4.7 years, age range 66–79) analyzed by intracellular immunofluorescence staining following stimulation of the BMMC population with PMA and ionomycin. *N* = 10 in each group. Data were obtained from five independent experiments with two samples each and shown as mean ± SEM in the graphs. Unpaired *t* test, ^*^ *p* = 0.02. (H) ROS levels ( = DHE MFI) in BMMCs in correlation with age. Spearman coefficient (*r*_s_), *p* value and sample size (*N*) are shown in the graph. (I) ROS levels in PBMCs and BMMCs. Paired *t* test, *N* = 9 in each group, age 67 ± 9.2, range 52–80. ^**^ *p* = 0.002. The bars represent mean ± SEM. Data were collected in five experiments with 1–2 samples each.

Proinflammatory molecules are believed to accumulate with age throughout the body inducing a basal level of inflammation that may contribute to age‐related diseases 36. We aimed to assess whether the production of proinflammatory cytokines other than IL‐15 and IL‐6 also increases in the BM with age. We therefore analyzed mRNA expression of IFN‐γ (Fig. 4A) and TNF (Fig. 4B) in BMMCs. mRNA levels of both inflammatory cytokines correlated with age, although the correlation between IFN‐γ and age was relatively weak. Some old donors had a high expression of the two molecules while in younger persons mRNA levels were frequently just over the detection limit or undetectable. In nine donors, the age‐related increase in the expression of both proinflammatory cytokines was confirmed in BMMC supernatants using ELISA (Fig. 4C, D).

CD8^+^CD28^−^ T cells are enriched in the aged BM (Fig. 4E and 34). Since this subpopulation is known to contribute to age‐related inflammation 33, 37, we analyzed the production of IFN‐γ‐ and TNF within the CD8^+^CD28^−^ T‐cell population in a group of younger (< 60 years) and in a group of old (>65 years) donors, comparing it with the expression of the same molecules in CD8^+^CD28^+^ and CD4^+^ T‐cell subpopulations (Fig. 4F–G). High percentages of CD8^+^CD28^−^ T cells expressed IFN‐γ‐ and TNF. No age‐related differences in the percentage of cytokine expressing cells were found among CD8^+^CD28^−^ and CD4^+^ T cells. More CD8^+^CD28^+^ T cells from the older group expressed IFN‐γ compared to the younger group (*p* = 0.02) while no difference in the expression of TNF was found.

Aging per se as well as proinflammatory molecules have been shown to influence ROS levels, contributing to oxidative stress 38, 39. In line with the increased levels of IFN‐γ and TNF in the BM with age and the high percentage of IFN‐γ‐ and TNF‐producing CD8^+^CD28^−^ BM cells, we also expected higher ROS levels in the proinflammatory BM environment of old donors. We therefore measured ROS in BMMCs from 20 donors with an age range from 40 to 87 years (Fig. 4H). There was a highly significant correlation between ROS and age. In addition, ROS levels were elevated in BMMCs compared to PBMCs from the same donors (Fig. 4I). These findings demonstrate that oxidative stress is associated with elevated levels of proinflammatory cytokines in the aged BM.

### ROS levels correlate with IL‐15 and IL‐6 expression in BMMCs

**Figure 5:**
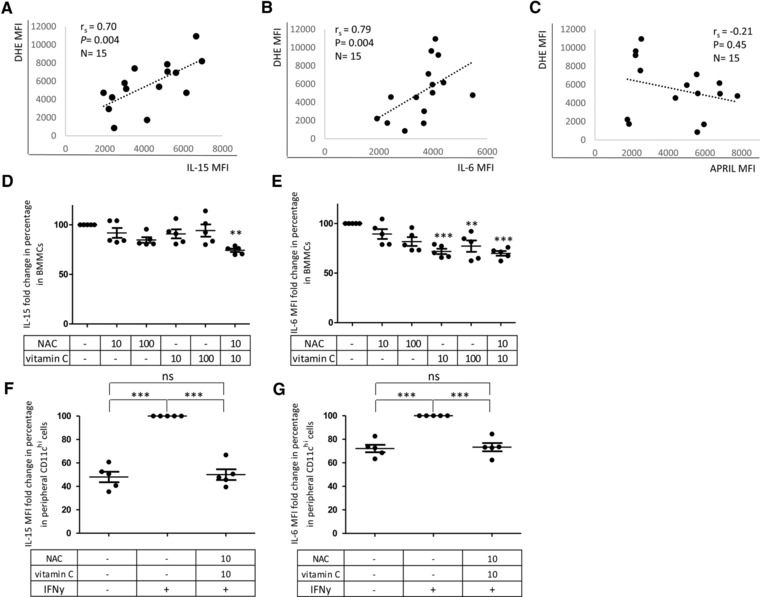
ROS correlates with IL‐15 and IL‐6 but not with APRIL in BMMCs and ROS scavenging reduces IL‐15 and IL‐6 expression. ROS were measured by dihydroethidium (DHE) MFI as described in Materials and methods. IL‐15, IL‐6, and APRIL were assessed by immunofluorescence staining. Correlations between ROS levels and IL‐15 MFI (A), IL‐6 MFI (B), and APRIL MFI (C) in BMMCs are shown. Age 66 ± 10.7 years, range 48–82. Spearman coefficient (*r*_s_), *p* value and sample size (*N*) are shown in each graph. Data were collected in seven experiments performed with 1–2 samples. (D) IL‐15 and (E) IL‐6 reduction in percent after incubation with NAC and vitamin C expressed as MFI following immunofluorescence staining and FACS analysis. Unstimulated BMMCs were incubated in the presence or absence of NAC and 10 μM or vitamin C or a combination of NAC and vitamin C. MFI values of each cytokine were normalized against MFI values of untreated controls, indicated in the graph as 100%. *N* = 5 in each experimental condition. Age 67 ± 6.8 years, range 58–75 in each group. One way ANOVA, Bonferroni post hoc test. Significances relative to the untreated controls are reported. ^**^ *p* < 0.01, ^***^ *p* < 0.001. (F) IL‐15 and (G) IL‐6 reduction in percentage in peripheral CD11c^hi^ cells after incubation with NAC and vitamin C in the presence of IFN‐y within PBMCs. IFN‐y‐treated cells in the absence of ROS scavengers are considered as 100%. PBMCs were incubated with or without IFN‐γ with or without a combination of NAC and vitamin C. *N* = 5 for each experimental condition. Age 37 ± 5 years, range 32–45 in each group. One way ANOVA, Bonferroni post hoc test. Significances relative to the comparisons between treatments are shown. ^***^ *p* < 0.001. The bars represent mean ± SEM. Data were obtained from five independent experiments with one donor each.

To analyze the impact of oxidative stress on the maintenance of immunological memory in the BM, we investigated whether ROS correlated with effector/memory cell survival factors in BMMCs. We show that IL‐15 and IL‐6, but not APRIL expression correlated with ROS levels in BMMCs (Fig. 5A–C). To confirm that ROS also play a causal role in regulating the expression of IL‐15 and IL‐6, unstimulated BMMCs were incubated with the ROS scavengers NAC and vitamin C at different concentrations (Fig. 5D–E). While NAC or vitamin C alone did not change IL‐15 expression, the combination of 10 μM NAC and 10 μM vitamin C significantly downregulated IL‐15 compared to untreated controls (Fig. 5D). IL‐6 was downregulated by vitamin C alone as well as by the combination of NAC and vitamin C (Fig. 5E).

In order to model the situation in the human BM in vitro, we tested how well IL‐15 and IL‐6 could be stimulated in CD11c^hi^ cells by IFN‐y and whether a potential effect could be blocked by ROS scavenger molecules. This cell type was selected because of its characteristically high production of both cytokines in the BM in old age (Fig. 3A, D). IL‐15 and IL‐6 expression in CD11c^hi^ cells did not change when BMMCs were stimulated with IFN‐y (Supporting Information Fig. 3). In view of the increased sensitivity to stimulation with cytokines and to the lower ex vivo ROS levels compared to BMMCs, we decided to perform this experiment using PBMCs. We stimulated PBMCs for 2 days with 10 ng/mL IFN‐y in the presence or absence of the ROS scavengers NAC and vitamin C and we quantified IL‐15 and IL‐6 expression by FACS analysis in CD11c^hi^ cells (Fig. 5F, G). Stimulation with IFN‐γ was found to significantly upregulate the expression of IL‐15 and IL‐6 in peripheral CD11c^hi^ cells. Treatment of IFNγ‐stimulated PBMCs with 10 μM NAC and 10 μM vitamin C induced a reduction of IL‐15 and IL‐6 in CD11c^hi^ cells leading to cytokine levels similar to the ones observed in cells cultured in the absence of IFN‐γ. In addition, we demonstrated the intracellular localization of IL‐15 and IL‐6 using the ImageStream system. Again, we used peripheral CD11c^hi^ cells, which were either unstimulated or stimulated with 10 ng/mL IFN‐γ for 2 days. The IFN‐γ‐mediated induction of IL‐15 and IL‐6 in the cytoplasm of CD11c^hi^ cells was clearly visible (Supporting Information Fig. 4).

Taken together, our results on human BM and PBMCs suggest that ROS may play an important role for age‐related changes of BM niches, particularly on an inflammatory background. This suggests a role of oxidative stress in regulating the production of survival molecules for CD8^+^ effector cells in the aged BM, presumably leading to the accumulation of CD8^+^CD28^−^ T cells in the BM in old age.

### Accumulation of IL‐15 and IL‐6 in the BM of superoxide dismutase 1 (SOD1)^−/−^ mice

**Figure 6:**
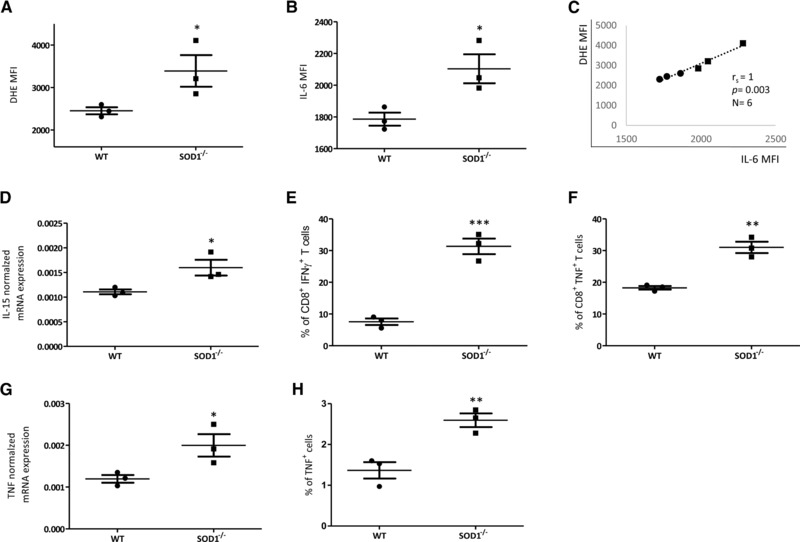
ROS and proinflammatory cytokines are increased in the BM of SOD1^−/−^ mice. (A) ROS levels assessed by DHE fluorescence staining and (B) IL‐6 also analyzed by immunofluorescence staining and FACS analysis (MFI) in the BM of SOD1^−/−^ mice and WT mice. Unpaired *t* test, comparison between groups, ^*^ *p* < 0.05. (C) ROS levels in correlation with IL‐6 MFI in BM cells from WT and SOD1^−/−^ mice. Spearman coefficient (*r*_s_), *p* value and sample size (N) are shown in the graph. (D) IL‐15 mRNA expression analyzed in BM cells by qPCR. The values are normalized against the housekeeping gene β‐actin. Unpaired *t* test, comparison between groups ^*^ *p* < 0.05. Percentage of (E) IFN‐γ‐ and (F) TNF‐producing CD8^+^ T cells in the BM of SOD1^−/−^ and WT mice. The total CD8^+^ T‐cell population is considered 100%. Cells were analyzed by immunofluorescence staining. Intracellular IFN‐γ and TNF were measured in BM cells following stimulation with PMA and ionomycin. TNF mRNA (G) and protein (H) in BM cells from WT and SOD1^−/−^ mice. Unpaired *t* test, comparison between groups ^***^ *p* < 0.001, ^**^ *p* < 0.01, *N* = 3 in each group from a single experiment.

To obtain final evidence for the importance of ROS in stimulating the production of IL‐15 and IL‐6 and the accumulation of CD8^+^ effector T cells in the BM, we used a mouse model. The expression of both cytokines was quantified in the BM of SOD1^−/−^ and WT mice (Fig. 6). As expected, ROS levels were higher in the BM of SOD1^−/−^ mice compared to their WT counterparts (Fig. 6A). In addition, IL‐6 was overexpressed in the BM from SOD1^−/−^ mice (Fig. 6B). In accordance with our previous data in human cells, there was a high correlation between ROS and IL‐6 levels in the BM of SOD1^−/−^ mice as well as in WT controls (Fig. 6C). We measured IL‐15 mRNA using quantitative PCR (qPCR) due to a lack of a suitable antibody. IL‐15 mRNA levels were increased in the BM of SOD1^−/−^ compared to WT littermates (Fig. 6D). We then analyzed whether the number of IFN‐γ‐ and TNF‐producing CD8^+^ T cells was also increased in the BM of SOD1^−/−^ mice. There were significantly more IFN‐γ‐ and TNF‐producing CD8^+^ T cells in the BM of SOD1^−/−^ mice in comparison to the BM of WT mice (Fig. 6E and F). In addition, higher TNF mRNA and more TNF‐expressing BM cells were found in SOD1^−/−^ mice (Fig. 6G, H). These results in mice confirm our concept that high ROS levels influence the BM cytokine production pattern, leading to an overexpression of IL‐15 and IL‐6 and to the accumulation of IFN‐γ‐ and TNF‐producing CD8^+^ T cells as a result.

## Discussion

Aging has a negative effect on the quality of immune responses, which increases the frequency and severity of infectious diseases and reduces the result of vaccinations 40. Innate as well as adaptive immunity deteriorates, but T lymphocytes are most severely affected, as they lose their maturation organ, the thymus 1, 2. Thymic involution leads to a gradual loss of naïve T cells combined with an increase in the proportion of antigen‐experienced cells. These changes are particularly pronounced in the CD8^+^ T‐cell pool as well as in persons with persistent viral infections such as with CMV 33, 41, 42.

Special attention has been given to the fact that CD8^+^CD28^−^ effector T cells accumulate in old age and in patients with latent CMV infection 33. These cells are proinflammatory, as they produce large amounts of IFN‐γ and TNF, fail to proliferate upon antigenic stimulation, and have short telomeres 43. Because of the latter characteristic, CD8^+^CD28^−^ T cells are frequently referred to as “senescent” cells. They are also a parameter of the so‐called Immune Risk Profile (IRP) that goes along with an increased mortality rate in old persons 32, 44. This is one of the reasons why CD8^+^CD28^−^ T cells are generally believed to represent a potentially harmful component of the aging immune system. As highly differentiated effector cells, they are prone to undergo apoptosis easily but can be rescued by IL‐15 35. Among other organs, IL‐15 is produced in the BM where we have shown IL‐15‐producing stroma cells in close vicinity to CD8^+^ T cells 9. CD8^+^CD28^−^ T cells also accumulate in the BM that prompted us to hypothesize that there they may occupy space otherwise available for long‐lived plasma cells and CD4^+^ helper T cells 40. Whether the survival and accumulation of CD8^+^CD28^−^ T cells in the BM is due to intrinsic changes of the cells themselves or to a restructuring of BM niches with age is not yet known.

We investigated the possibility that survival factors in the BM change with aging. We found that IL‐15 and IL‐6 increase while IL‐7 and APRIL decrease with age. CXCL‐12, a chemokine required for the recruitment of cells to the plasma cell niche, was not affected by aging. These data are in favor of the possibility that changes in the production spectrum of survival factors in the BM lead to a loss of plasma cells and CD4^+^ helper cells, in contrast to an accumulation of highly differentiated CD8^+^ T cells 34, 45. In spite of the fact that IL‐15 as well as IL‐6 has multiple functions, they are both also known as proinflammatory molecules 46, 47. It is therefore tempting to speculate that age‐related changes in the production of immune cell survival factors in the BM are the result of the chronic subclinical inflammatory state associated with old age referred to as “inflamm‐aging” 36. This possibility is supported by our results that IFN‐γ and TNF mRNA were increased in the BM in old age and that the number of IFN‐γ‐producing CD8^+^CD28^−^ T cells was high. IFN‐γ is likely to activate cells of the myeloid lineage. In accordance with this fact we show that IFN‐γ stimulates IL‐15 as well as IL‐6 production by CD11c^hi^ cells in vitro and there is also a correlation between age and IL‐15 in CD11c^hi^ and of IL‐6 in CD11c^hi^ and CD14^+^ cells in the BM.

Inflammatory processes are therefore important for age‐related remodeling of BM niches. This raises the question: what triggers inflammation in this specific organ in old age? It is well understood that age‐related oxidative stress is associated with chronic inflammation, and there have been frequent links between oxidative stress, “inflamm‐aging” and immunosenescence 48, 49. A recent article on mitochondrial dysfunction and increased ROS production as a cause of DC dysfunction in old age is also in favor of this possibility 39. We show that ROS production also increases in BMMCs with age and that ROS levels are significantly higher in BMMCs than in PBMCs. ROS also correlate with IL‐15 and IL‐6 but not with APRIL in the BM, suggesting that there might be a link between the production of these molecules.

**Figure 7:**
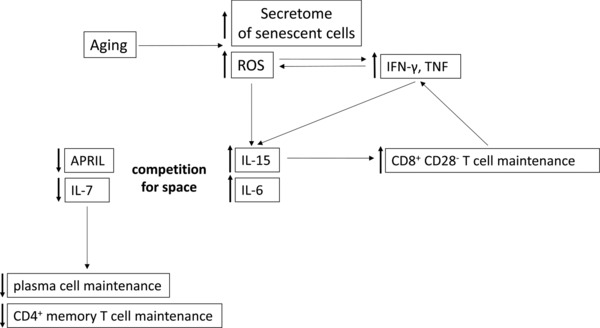
Schematic representation of the links between aging, ROS, inflammatory cytokines, and adaptive immune cell survival factors. Aging leads to changes in the mitochondria. This results in increased ROS production. Senescent cells characteristically have a secretome with inflammatory properties. Both factors can contribute to increased IL‐15 and IL‐6 production in the aged BM. These two cytokines synergize in the generation and maintenance of CD8^+^ CD28^−^ T cells, which produce IFN‐γ and TNF, which can again stimulate the production of ROS. IL‐6‐ and IL‐15‐producing cells may occupy space otherwise reserved for cells capable of APRIL and IL‐7 production, which would normally be responsible for plasma cell and CD4^+^ memory cell maintenance.

Whether ROS stimulate the production of proinflammatory cytokines or whether inflammation triggers oxidative stress is not clear, but most likely both mechanisms are operative in the BM during aging (Fig. 7) 38. Our results on the decrease of IL‐15 and IL‐6 production by ROS scavengers in human cells underline the importance of oxidative stress, possibly in the first place triggered by the aging process 50. Our data on SOD1^−/−^ mice that have increased IL‐15 and IL‐6 concentrations as well as an accumulation of IFN‐γ‐ and TNF‐producing T cells in the BM further support this point.

We thus propose the following concept (Fig. 7): cellular aging in the BM may induce mitochondrial damage, increase ROS production and trigger the production of an inflammatory secretome by senescent cells 51. These factors all induce IL‐15 and IL‐6 that results in the accumulation of IFN‐γ‐ and TNF‐producing CD8^+^CD28^−^ T cells. IFN‐γ and TNF further stimulate ROS as well as IL‐15 and IL‐6 production, thus leading to a vicious circle. Whether the observed decrease of IL‐7 and APRIL and increase of IL‐15 and IL‐6 represent the result of a competition for space of different BM stroma cells or whether there is a direct interaction between different cell types/cytokines is not yet clear, but will be the subject of further investigations.

Our data allow the cautious assumption that treatment with antioxidants may be helpful in keeping inflammation levels in the BM low and ensuring sufficient production of the “right” memory cell survival factors. Maintenance of immunological memory in old age may thus be improved.

## Materials and methods

### Human sample collection and preparation

Human BM and blood samples were obtained from systemically healthy individuals who did not receive immunomodulatory drugs or suffer from diseases known to influence the immune system, including autoimmune diseases and cancer. Hip replacement surgery was performed and bone from the femur shaft was harvested. A biopsy of substantia spongiosa osseum, which would otherwise have been discarded, was used to isolate BMMCs. BM biopsies were fragmented, washed once with complete RPMI medium (RPMI 1640 supplemented with 10% FCS, 100 U/mL penicillin, and 100 μg/mL streptomycin; Invitrogen) and treated with purified collagenase (CLSPA, Worthington Biochemical; 20 U/mL in complete RPMI medium) for 1 h at 37°C. BM biopsies were then centrifuged and BMMCs purified by density gradient centrifugation (Ficoll‐Hypaque). Experiments were performed using BMMCs and not with all BM cells in order to obtain cleaner populations and thus to improve the quality of the results. qPCR experiments comparing the expression of effector/memory cell survival factors in BMMCs and in BM cells showed similar results (data not shown). PBMCs were used for pilot and stimulation control experiments. Purification of PBMCs from heparinized blood was also performed by density gradient centrifugation.

### Mice

Six‐month‐old male SOD1^−/−^ mice on a C57BL/6J genetic background 52 were bred under specific pathogen‐free conditions at the Institute for Molecular Biotechnology of the Austrian Academy of Sciences in Vienna, Austria. For all experiments littermate controls were used. BM cells were obtained from mice by flushing the femur and tibia with PBS.

### Isolation of RNA and quantitative RT‐PCR

RNA was isolated from purified BMMCs using the RNeasy Plus mini kit (Qiagen). First‐strand cDNA synthesis was performed using a Reverse Transcription system (Promega). qRT‐PCR experiments were performed using the LightCycler 480 System (Roche Diagnostics), 2X SYBR Green 1 Master (Roche Diagnostics), and β‐actin as housekeeping gene for relative quantification of effector/memory cell survival factors. Sequence‐specific oligonucleotide primers were designed using Primer3 software 53 and synthesized by MWG Biotech (Ebersberg, Germany). The primer sequences are listed in Supporting Information Table 1.

### Cell culture

Incubation times were chosen based on pilot experiments on time courses. PBMCs were stimulated for 2 days in complete RPMI with 10 ng/mL IFN‐γ in the presence or absence of 10 μM NAC and 10 μM vitamin C (Sigma Aldrich). BMMCs were also incubated for 2 days in complete RPMI at 37°C in the presence or absence of NAC and vitamin C at different concentrations. The last 4 h of stimulation were performed in the presence of 10 mg/mL brefeldin A (BFA; Sigma‐Aldrich).

### Flow cytometric analysis

To analyze the expression of effector/memory cell survival factors, BMMCs were incubated with 10 mg/mL BFA for 15 h at 37°C. The production of cytokines by CD28^−^ T cells in the BM was measured after stimulating the cells for 4 h with 30 ng/mL PMA and 500 ng/mL ionomycin in the presence of 10 mg/mL BFA. Immunofluorescence surface staining was performed by adding a panel of directly conjugated Abs to freshly prepared PBMCs and BMMCs. Dead cells were excluded from the analysis using a fixable viability dye (Zombie Aqua™ Fixable Viability Kit, Biolegend). After surface staining, cells were permeabilized using the Cytofix/Cytoperm kit (BD Pharmingen), and incubated with intracellular Abs. Labeled cells were measured by a FACSCanto II (BD Biosciences). Data were analyzed using Flowjo software. The following labeled Abs were used: hCD11c‐PE (S‐HCL3) and hCD11c‐APC (S‐HCL3) were purchased from BD. hCD8‐PerCP (5K1), hCD14‐PeCy7 (Sa14‐2), hCD34‐PerCP (581), and mIFN‐γ‐PE (XMG1.2) were purchased from Biolegend. hCD3‐BV510 (UCHT1), mCD8‐PerCP (53‐6.7), hCD28‐PeCy7 (CD28.2), hIL‐6‐APC (MQ2‐13A5), hIFN‐γ‐FITC (XMG1.2), and mIL‐6‐FITC (MP5‐20F3) were purchased from ebioscience. hIL‐15‐APC (34559) and hCXCL‐12‐APC (79018) were purchased from R&D Systems. hAPRIL‐APC (MAB8843) was purchased from Novus Biologicals.

### Ab specificity tests

Each Ab was carefully titrated before being used for the experiments. The specificity of hIL‐15 and hIL‐6 Abs was assessed by preincubating each Ab with 1 ng/mL of recombinant IL‐15 or IL‐6 (Immunotools) for 1 h at 4°C (data not shown). hIL‐15 Ab was additionally tested using cell lines expressing (SGBS preadipocytes) or not expressing (Jurkat cells) the cytokine (Supporting Information Fig. 5). Cells not expressing the antigen did not give any staining. SGBS cells were stimulated for 24 h with 10 ng/mL IFN‐γ and the expression of IL‐15 was measured at the mRNA (Supporting Information Fig. 5C) and at the protein level (Supporting Information Fig. 5D).

Autofluorescence of CD11c^hi^, CD14^+^, and CD34^+^ cells was excluded with control experiments (Supporting Information Fig. 6).

### ELISA assay

BMMCs were seeded at a density of 5 × 10^6^ cells, cultured for 5 h in complete RPMI in the presence or absence of 30 ng/mL PMA and 500 ng/mL ionomycin and supernatants were collected. IL‐6 and APRIL were measured in the supernatants from unstimulated BMMCs while IFN‐γ and TNF were assessed in supernatants from BMMCs stimulated with PMA and ionomycin using ELISA kits (R&D Systems).

### ImageStream

PBMCs were incubated in the presence or absence of 10 ng/mL IFN‐γ for 2 days. Intracellular production of IL‐15 and IL‐6 in CD11c^hi^ cells was measured using ImageStream Mark II (MKII) Imaging Flow Cytometer system (Amnis). Cells were gated on aspect ratio versus bright field area to include only single cells, using the gradient root‐mean‐square feature to include cells in focus. Apoptotic cells were excluded from the analysis based on morphology. Cells in focus were gated for the expression of CD11c and cytokine expression was analyzed using IDEAS v6.0340.0 software.

### ROS measurement

ROS levels were measured after incubation of BMMCs and PBMCs with the fluorescent dye dihydroethidium (Sigma‐Aldrich) at a concentration of 1:250 in complete RPMI for 20 min at 37°C. The surface Abs CD11c and CD14 were added at the same time.

### Statistical analysis

Statistical significance was assessed by Spearman correlation analysis, one way ANOVA with Bonferroni post hoc test and unpaired or paired two‐tailed t test, as indicated in the figure legends. A *p* value less than 0.05 was considered significant.

### Study approval

Human studies were approved by the Ethics Committees of the “Klinikum Wels‐Grieskirchen” (Austria) and of the Innsbruck Medical University (Austria). Written informed consent was received from participants prior to inclusion in the study.

Animal experiments were carried out in accordance with the Austrian law for animal protection and in accordance with institutional guidelines.

## Conflict of interest

The authors declare no commercial or financial conflict of interest.

**Abbreviations**
APRIL: a proliferation‐inducing ligand
BM: bone marrow
BMMC: BM mononuclear cell
hi: high
NAC: N‐acetylcysteine
SOD1: superoxide dismutase 1

## Supporting information

SupportingInformation Figure1
SupportingInformation Figure2
Supporting Information Figure 3
SupportingInformation Figure4
Supporting Information Figure 5
SupportingInformation Figure6
SupportingInformation Table 1

SupportingInformation
