# Supplementary material for: “Inflamm‐aging” influences immune cell survival factors in human bone marrow
Source: Eur J Immunol. 2017 Jan 11;47(3):481–92. doi: 10.1002/eji.201646570 (PMC5434810; doi:10.1002/eji.201646570)
Supplement: Supplementary file 1 — SupportingInformation Figure1 SupportingInformation Figure2 Supporting Information Figure 3 SupportingInformation Figure4 Supporting Information Figure 5 SupportingInformation Figure6 SupportingInformation Table 1 [file EJI-47-481-s001.pdf]

European Journal of Immunology

Supporting Information
for
DOI 10.1002/eji.201646570

Luca Pangrazzi, Andreas Meryk, Erin Naismith, Rafal Koziel, Julian Lair, Martin Krismer, Klemens Trieb and Beatrix Grubeck-Loebenstein

“Inflamm-aging” influences immune cell survival factors in human bone marrow

© 2016 The Authors. *European Journal of Immunology* published by WILEY-VCH Verlag GmbH & Co. KGaA, Weinheim.

Supporting Information Figure 1

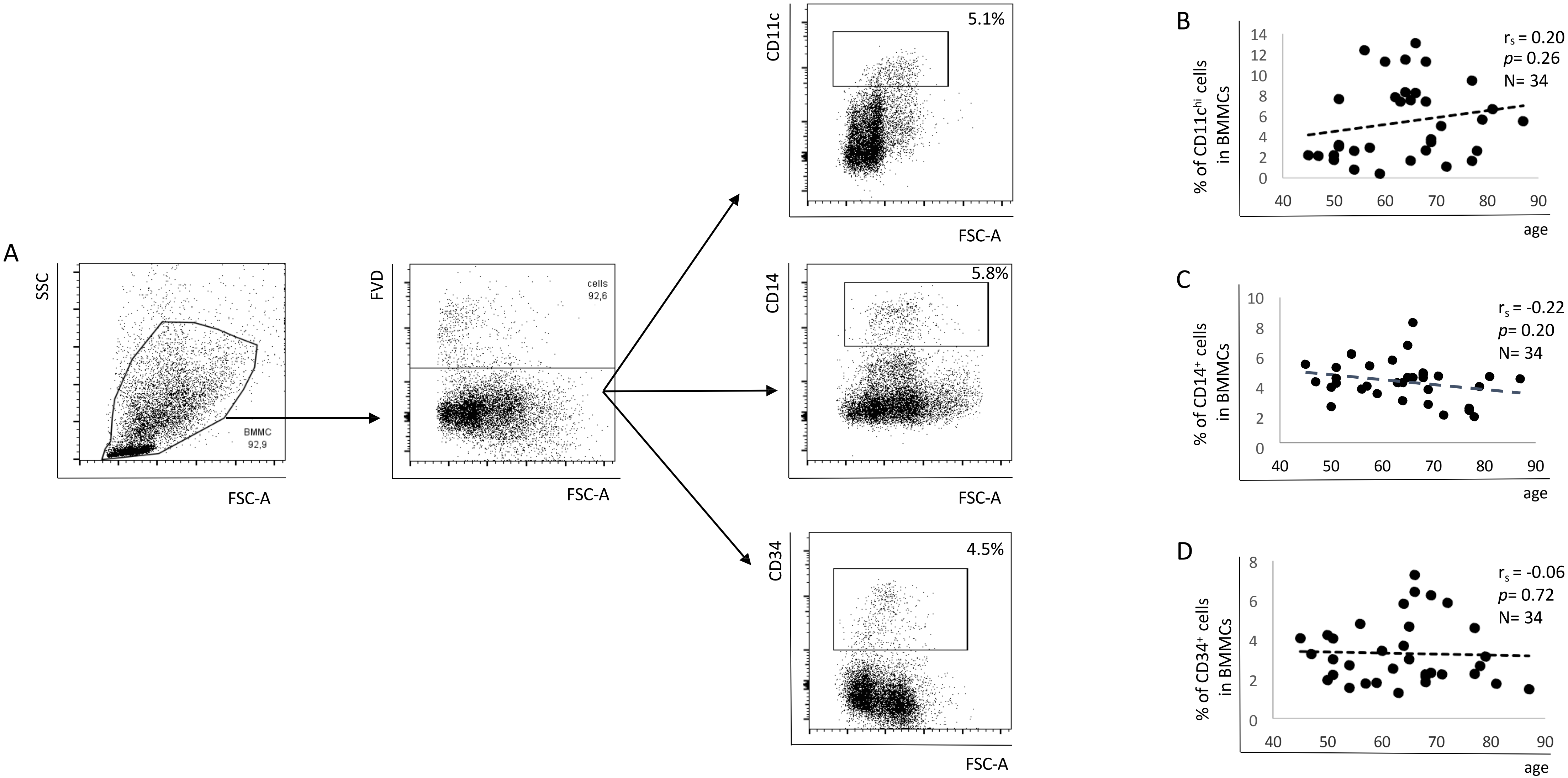

Subpopulations in BMMCs likely to produce effector/memory cell survival factors. A) The gating strategy by FACS analysis is shown. After excluding dead cells, BMMCs expressing CD11c^{hi}, CD14 and CD34 were gated for further analysis. Percentages of (A) CD11c^{hi} (B), CD14^{+} (C) and CD34^{+} cells (D) in BMMCs (=100%) in correlation with age are shown. Spearman coefficient (r_s), p value and sample size (N) are shown in each graph.

Supporting Information Figure 2

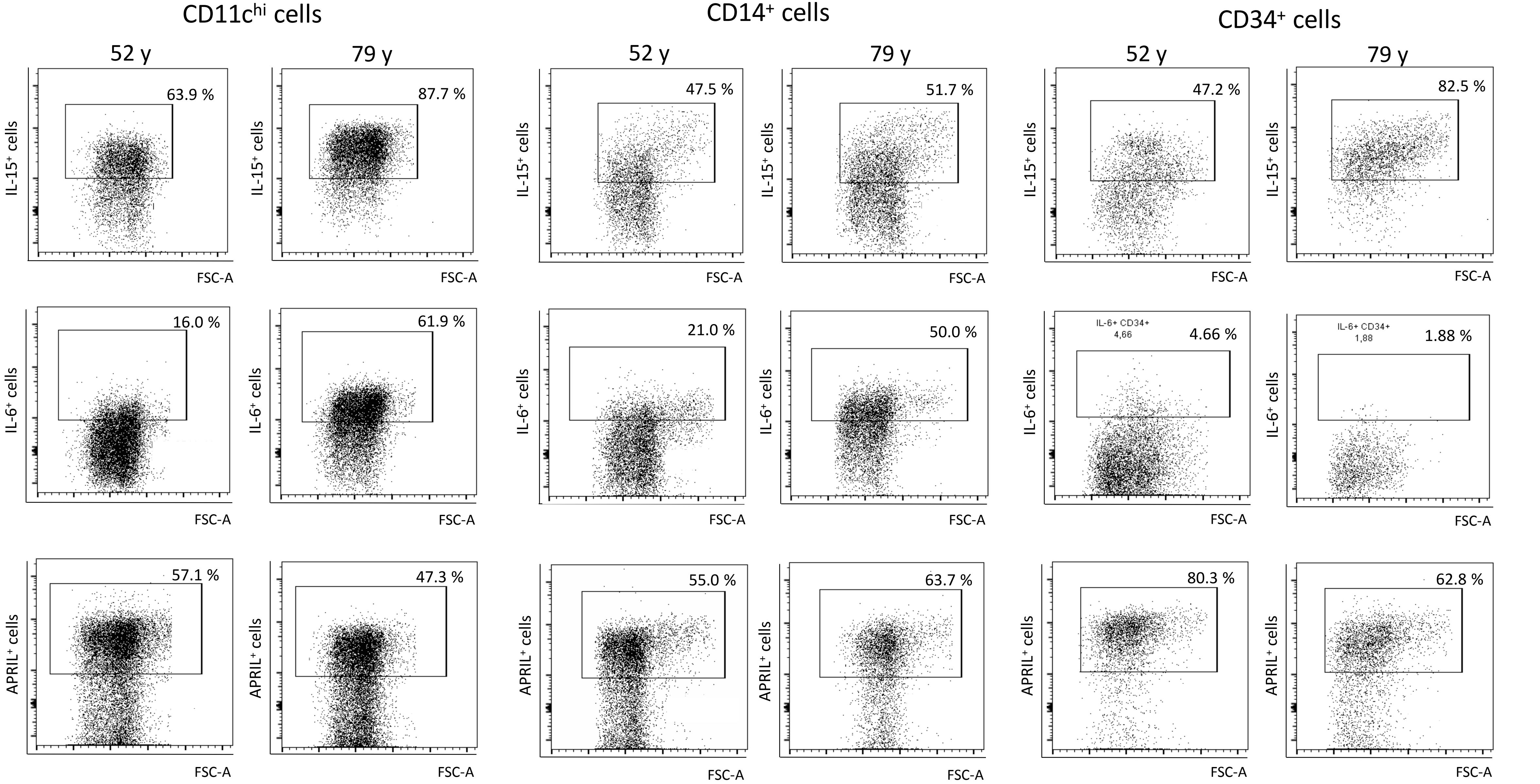

Dot plots for IL-15, IL-6 and APRIL in CD11c^{hi}, CD14$^+$ and CD34$^+$ cells in one representative younger (52 years) and one old donor (79 years). Percentages of cells expressing the respective cytokines are shown in each dot plot. CD11c^{hi}, CD14$^+$ and CD34$^+$ cells are gated as shown in Suppl.Fig. 1 and the respective cell type is always considered as 100%.

Supporting Information Figure 3

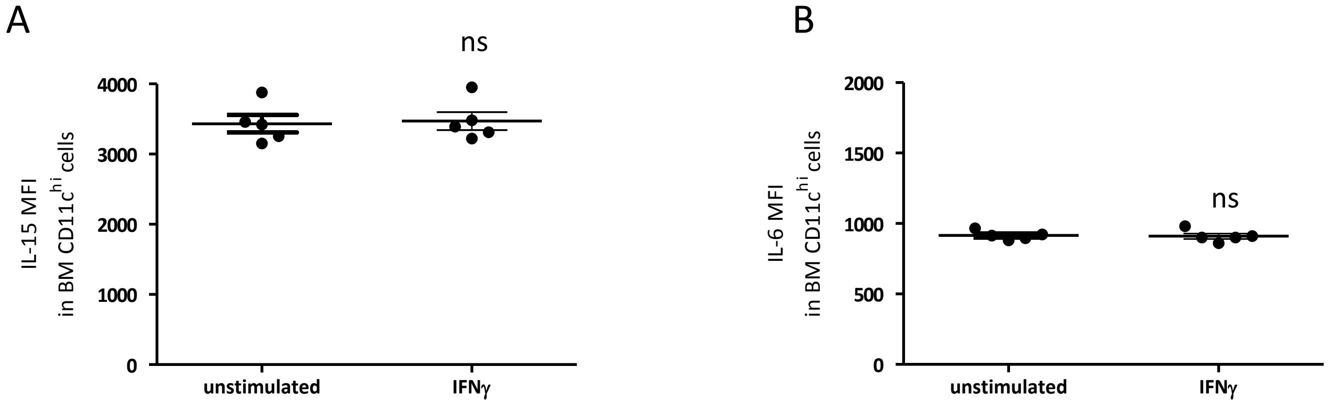

Stimulation of BMMCs with IFN-γ (10 ng/ml) does not increase IL-15 (A) and IL-6 (B) expression in BM CD11c^{hi} cells. Paired t test, p=0.23 (IL-15) and p=0.65 (IL-6) . N=5, mean 67±6.8 years, age range 58-75 in each group. The bars represent mean ± SEM.

Supporting Information Figure 4

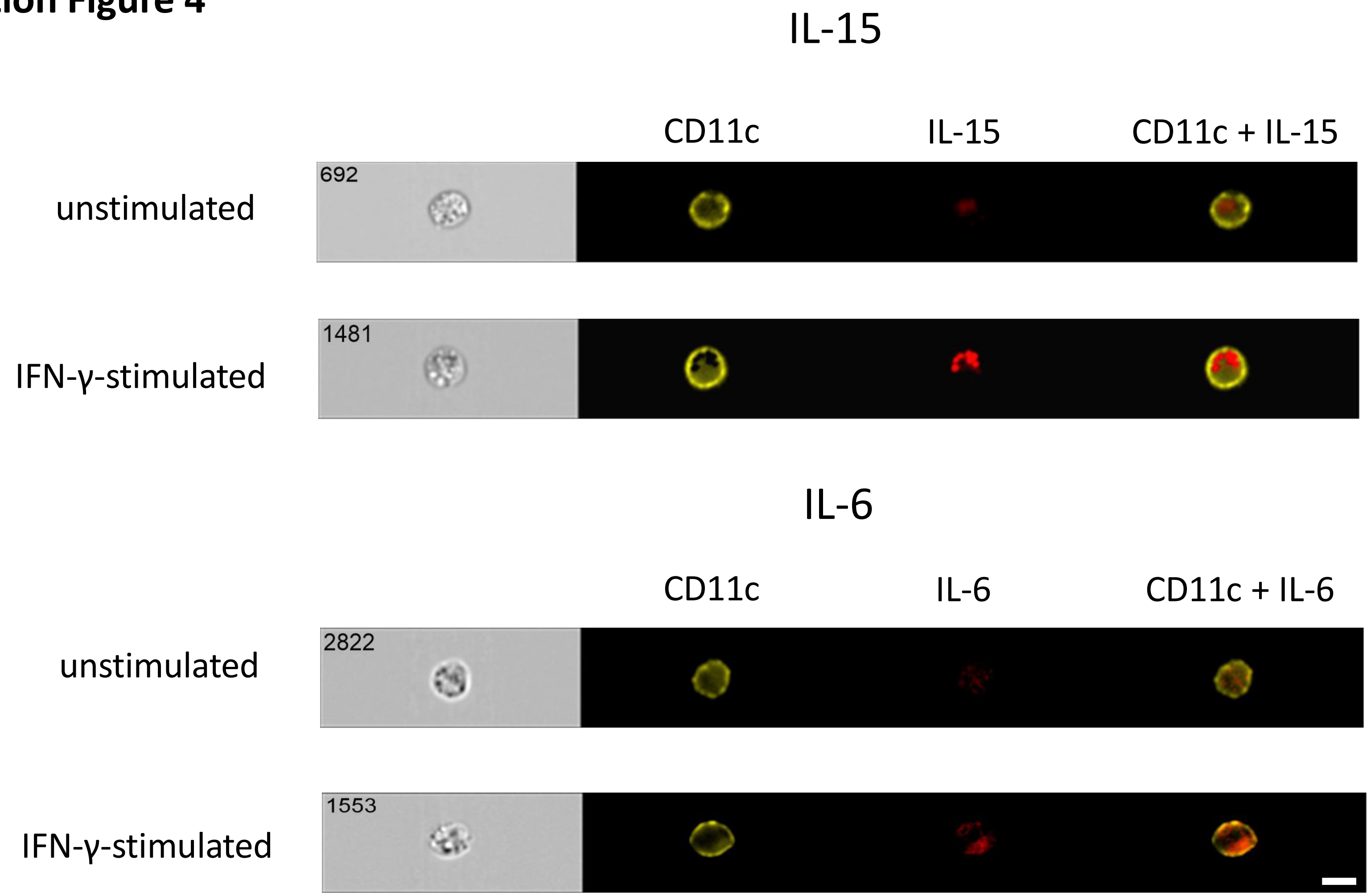

Demonstration of the intracellular localization of IL-15 and IL-6 in $CD11c^{hi}$ cells in PBMCs by ImageStream technique. Cells were either untreated or stimulated with IFNγ (10 ng/ml) for 2 days. They then underwent immunofluorescence staining and were analyzed by ImageStream (Amnis). Representative pictures with single stainings of CD11c, IL-15 and IL-6 and the combinations of CD11c+IL-15 and CD11c+IL-6 in stimulated and unstimulated samples are shown. Both cytokines are visible in the cytoplasm of unstimulated cells, but their expression increases after stimulation with IFN-γ. Scalebar = 5µm.

Supporting Information Figure 5

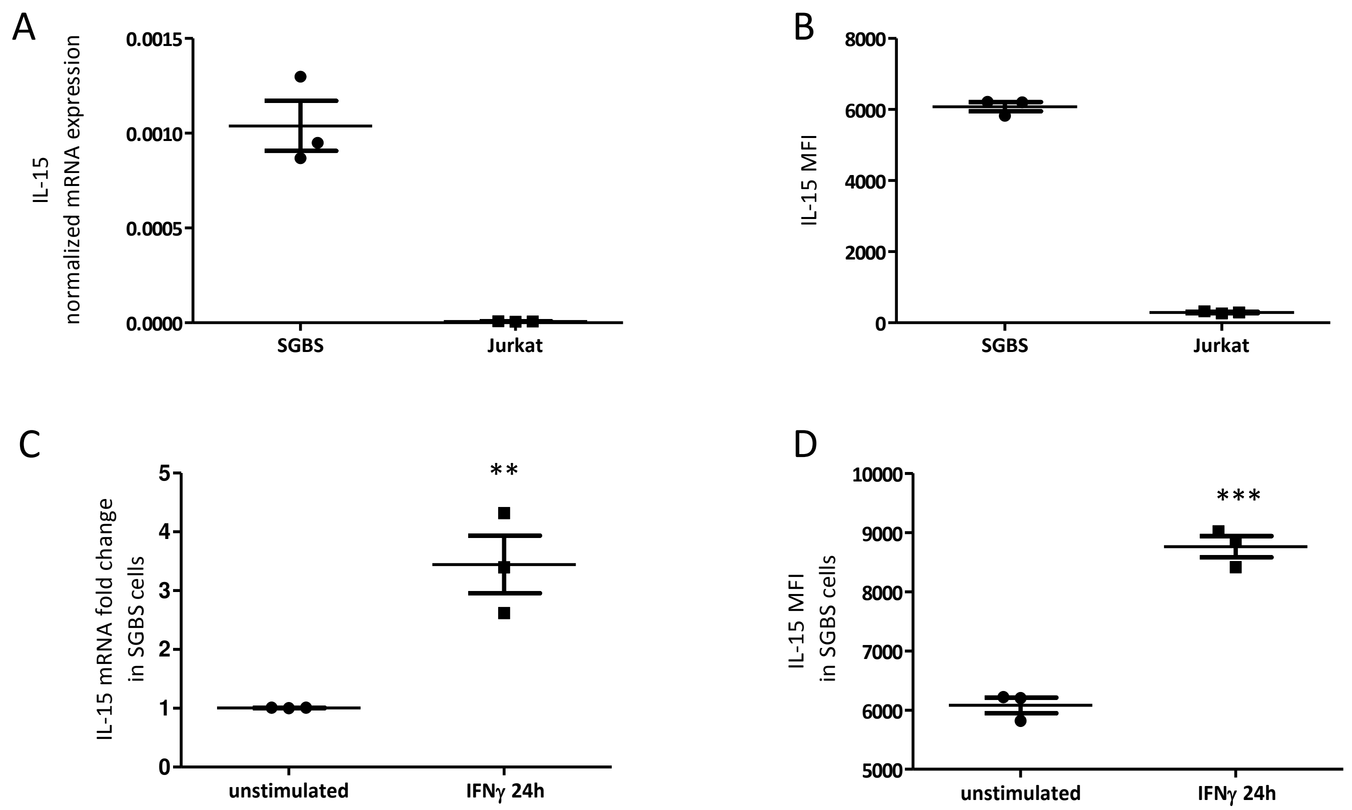

IL-15 expression in SGBS preadipocytes and Jurkat cells measured using qPCR (A) and FACS (B). IL-15 mRNA (C) and protein (D) expression in unstimulated SGBS cells and in cells stimulated for 24h with 10 ng/ml IFN-γ. Paired t test, ***p<0.001, **p<0.01, N=3. The bars represent mean ± SEM.

Supporting Information Figure 6

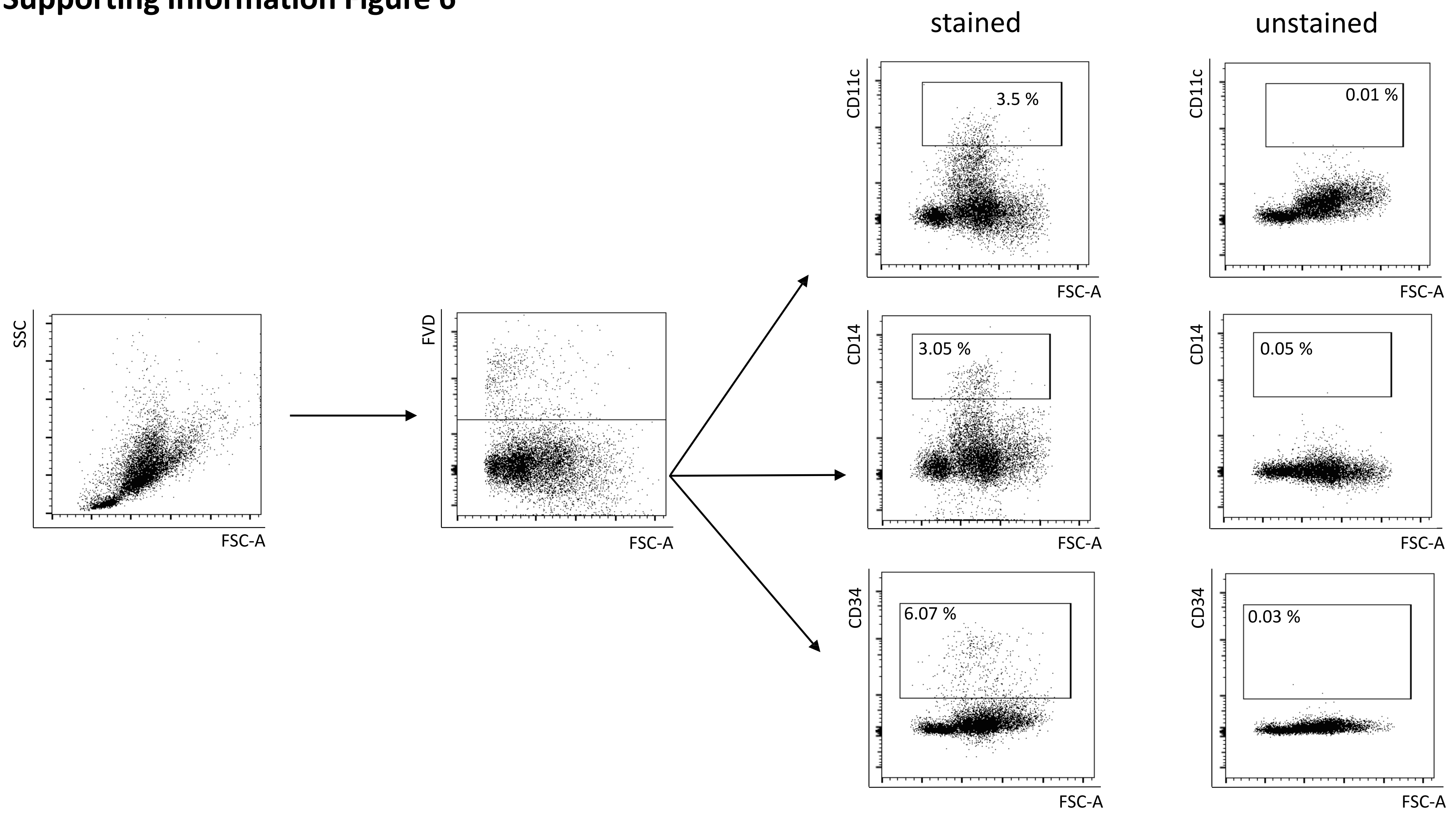

CD11c^{hi}, CD14^{+} and CD34^{+} cells are not autofluorescent cells. After removing dead cells from BMMCs, CD11c^{hi}, CD14^{+} and CD34^{+} subpopulations were gated. Unstained samples for CD11c, CD14 and CD34 cells respectively are shown in the Figure.

Supporting Information Table 1

Table 1: Sequences of forward and reverse primers used for qRT-PCR

	Forward primer (5'-3')	Reverse primer (5'-3')
hIL-15	ATTTTGGGCTGTTTCAGTGC	TTACTTTGCAACTGGGGTGA
hIL-7	GTAGCAATTGCCTGAATAATG	GTTGTGCCTTCTGAAACT
hIL-6	GGTACATCCTCGACGGCATCT	GTGCCT-CTTTGCTGCTTTCAC
hAPRIL	GAGACTCTATTCCGATGTATAA	CATGTGGAGAGAGGTTAAG
hCXCL-12	TGAGCTACAGATGCCCATGC	TTCTCCAGGTACTCCTGAATCC
hTNFα	GGAGAAGGGTGACCGACTCA	CTGCCCAGACTCGGCAA
hIFNγ	TGGAGACCATCAAGGAAGACA	GCGACAGTTCAGCCATCACT
hIL-1β	ACAGATGAAGTGCTCCTTCCA	GTCGGAGATTCGTAGCTGGAT
hβ ACTIN	TCCTCCCTGGGCATGGAGT	TCTCCTTCTGCATCCTGTCG
mIL-15	CATCCATCTCGTGCTACTTGTGTT	CATCTATCCAGTTGGCCTCTGTTT
mβ ACTIN	AGAGGGAAATCGTGCGTGAC	CAATAGTGATGACCTGGCCGT
