# Supplementary material for: “Inflamm‐aging” influences immune cell survival factors in human bone marrow
Source: Eur J Immunol. 2017 Jan 11;47(3):481–92. doi: 10.1002/eji.201646570 (PMC5434810; doi:10.1002/eji.201646570)
Supplement: Supplementary file 2 — SupportingInformation [file EJI-47-481-s002.pdf]

European Journal of Immunology

Supporting Information
for

DOI 10.1002/eji.201646570

Luca Pangrazzi, Andreas Meryk, Erin Naismith, Rafal Koziel, Julian Lair,
Martin Krismer, Klemens Trieb and Beatrix Grubeck-Loebenstein

“Inflamm-aging” influences immune cell survival factors in human bone marrow

© 2016 The Authors. *European Journal of Immunology* published by WILEY-VCH Verlag GmbH & Co. KGaA, Weinheim.

European Journal of Immunology

“Inflamm-aging” influences immune cell survival factors in human bone marrow

Luca Pangrazzi, Andreas Meryk, Erin Naismith, Rafal Koziel, Julian Lair, Martin Krismer, Klemens Trieb, Beatrix Grubeck-Loebenstein

Correspondence: Prof. Beatrix Grubeck-Loebenstein, Dept. of Immunology, Institute for Biomedical Aging Research, University of Innsbruck, Innsbruck, Austria

---

Review Timeline:	Submission date:	21-Jun-2016
	First Editorial decision:	15-Jul-2016
	Revision received:	21-Oct-2016
	Second Editorial decision:	16-Nov-2016
	Accepted:	14-Dec-2016

Handling Executive Committee member: Prof. Silvano Sozzani

Please note that the correspondence below does not include the standard editorial instructions regarding preparation and submission of revised manuscripts, only the scientific revisions requested and addressed.

First Editorial Decision
15-Jul-2016

Dear Prof. Grubeck-Loebenstein,

Manuscript ID eji.201646570 entitled "Inflamm-aging• influences immune cell survival factors in human bone marrow" which you submitted to the European Journal of Immunology has been reviewed. The comments of the referee(s) are included at the bottom of this letter.

A revised version of your manuscript that takes into account the comments of the referees will be reconsidered for publication. Should you disagree with any of the referees concerns, you should address this in your point-by-point response and provide solid scientific reasons for why you will not make the requested changes.

You should also pay close attention to the editorial comments included below. **In particular, please edit

European Journal of Immunology

your figure legends to follow Journal standards as outlined in the editorial comments. Failure to do this will result in delays in the re-review process.**

Please note that submitting a revision of your manuscript does not guarantee eventual acceptance, and that your revision will be re-reviewed by the referees before a decision is rendered.

If the revision of the paper is expected to take more than three months, please inform the editorial office. Revisions taking longer than six months may be assessed by new referees to ensure the relevance and timeliness of the data.

Once again, thank you for submitting your manuscript to European Journal of Immunology and we look forward to receiving your revision.

Yours sincerely,
Nadja Bakocevic

On behalf of
Prof. Silvano Sozzani

Dr. Nadja Bakocevic
Editorial Office
European Journal of Immunology
e-mail: ejied@wiley.com
www.eji-journal.eu

****

Reviewer: 1

Comments to the Author
In the present manuscript, Pangrazzi et al. analysed the impact of aging in the long-term maintenance of effector/memory T cells and plasma cells in the bone marrow (BM). In particular, they analyzed the production of survival factors (IL-7 and APRIL) as well as pro-inflammatory factors (IL-15 and IL-6) in BM mononuclear cells (BMMCs) from individuals of different ages. During aging, they observed an increase of IL-15 and IL-6, which correlates with the expansion of harmful CD8+ CD28+ senescent cells, and a decrease of IL-7 and APRIL, which are important survival factors for the maintenance of long-term effector/memory CD4+ T cells and plasma cells, respectively. They also correlated the production of IL-15

and IL-6 with IFN-γ and ROS levels, thus suggesting that anti-oxidants may improve immunological memory during aging. The results are interesting but key controls are missed and some experiments need a more extensive description.

Major points:

Figure 2. In this figure the authors evaluated the production of IL-15, APRIL, IL-6 and CXCL12 by intracellular staining of BMMCs. BMMCs are an heterogeneous population, thus measuring the level of cytokine production by intracellular staining is not informative, especially because in the Figure 3 they performed a deeper analysis of the BM cell types producing each cytokine. Instead, it is pivotal to demonstrate that all cytokines are secreted. Thus, the quantitative measurement of each cytokine in the culture supernatant of BMMCs from people of different ages is necessary.

Figure 3 and Supp. Fig.1: In figure 3, the authors show the intracellular staining of cytokine production performed in CD11chi, CD14+ and CD34+ BMMCs. An example of the gating strategies used to identify the three population is also shown in Supp. Fig.1. However, no data on the percentage of each population in all analysed individuals are shown. Moreover, the dot plots of the intracellular staining of all cytokines analysed in each population from at least a representative young and old individual should be shown in Supp Fig. 1.

Figure 4 C and D. In panel C, the authors show the percentage of IFN-γ and TN-α production in the CD8+ CD28- T cell population from the BMMCs of N=12 individuals. Several details are missed. Which is the age of the 12 individuals? How do they isolate CD8+ CD28- T cells for intracellular staining? Similar details are missed in the panel E that show ROS production in PBMCs and BMMCs from the same donors. Which donors? Are aged donors or young donors? Moreover, it is pivotal to compare: 1. The % CD8+ CD28- T cells producing IFN-γ and TNF-α between younger and older individuals; 2. The % of IFN-γ and TNF-α produced by CD8+ CD28-, CD8+ CD28+ and CD4+ T helper cells in both young and old individuals.

Figure 5. The authors correlate ROS production with cytokine expression in BMMCs (Panel A-C). Again, the details on the experimental procedure and population of BMMCs are missed. They reported the number of individuals (N=15) but not the range of age. In panels D to G, no details on the age of the individuals are reported. Moreover, in these experiments the authors used PBMCs instead BMMCs, "in view of the increased sensitivity of PBMCs to cytokines compared to BMMCs". In Figure 3, IL-15 and IL-6 are highly produced by CD11chi cells from BMMCs, thus it is not comprehensible why they decided to perform these experiments in PBMCs, which, as shown in Figure 4E, produce very lower levels of ROS compared to BMMCs. These experiments and those reported in Supp Figure 2 should be performed in CD11chi cells from BMMCs. Moreover, the authors correlate the accumulation of ROS with an

European Journal of Immunology

accumulation of CD8+ CD28- T cells. However, no data on the percentage of CD8+ CD28- T cells in BMMCs from young and old individuals are reported.

Finally, to correlate ROS accumulation with the survival of CD8+ CD28- T cells, these cells should be cultured in the presence or absence of NAC and Vit C and their survival rates should be evaluated.

Reviewer: 2

Comments to the Author

The paper by Pangrazzi et al. investigates the association between inflammation and ROS metabolism on the production of many factors that are important for the survival and differentiation of memory CD8+ T cells in the bone marrow. Most important, the authors analyze the impact of age. The manuscript reports some interesting observations, i.e. the association of age with the production of the proinflammatory molecules IL-15, IL-6 and IFN-g. This is not terribly surprising, as inflammaging• is known to occur in the elderly, however, analysis are performed in human bone marrow and for this reason are of some interest. Unfortunately, the paper lacks a main clear focus, is descriptive in most of its parts and appears fragmented. Some data are reported but are not thouroughly investigated, therefore it is difficult to assess the impact of some powerful correlations (e.g. APRIL) with the main message of the manuscript, i.e. memory CD8+ T cell survival. Finally, it is difficult for the reviewer to understand whether the focus is on total bone marrow memory CD8+ T cells, or on CD28“ terminally differentiated T cells, abundant in the elderly.

Major concerns:

1. the manuscript relies mostly on correlations. When correlating age with the production of proinflammatory proteins, the authors use flow cytometry to define the production of IL-6 and IL-15 by BMMC. In this regard, the authors normalize the data vs. the isotype control for every given sample. I have two major objections to this approach:

a. in the reviewer-γs experience measuring IL-15 by FACS in humans is tricky. Additional raw data should be provided to assess the validity of the assay. The authors mentioned they validated the antibody staining by pre-incubating the primary antibody with recombinant IL-15 or IL-6. While this demonstrates the specificity of the assay, it does not take into account the possibility that the staining with the primary antibody increases background fluorescence. Such a possibility can be assessed by carefully titrating the antibody on cell lines that express or not the antigen (not mentioned in Methods). Those cells not expressing the antigen (crossvalidated by qPCR for example or by western blot) should not give any staining. Staining with the isotype control does not give useful information on the unspecific binding of the antibody but only gives information on the possibility that the primary antibody binds Fc receptors.

b. As a consequence to point 1a, normalizing data vs. the isotype control is incorrect. Authors should

also consider that it is difficult to titrate isotypes and thus determine whether non-specific binding other than that to Fc receptors is occurring.

2. Could western blot of proinflammatory mediators be performed from BMMC lysates to confirm qPCR data?

3. In figure 3, APRIL MFI is inversely correlated with age only when measured on CD34+ cells. However, CD34+ cells, despite enriched, are a minor population in the BM. The same correlation is seen on total BMMC presented in Figure 1. Is it therefore important to show data on total BMMC if it is known that CD34+ cells drive the correlation? Moreover, evaluating the presence of a given antigen by FACS on total BMMC is difficult because BM harbours many different cell types with different autofluorescence. If those cells with the highest autofluorescence are the most abundant, the same cells will hide• the positivity of other cells and thus decrease sensitivity. This can be prevented by dedicated gating strategies. Unfortunately, not enough information is provided in this regard.

4. Figure 4c: authors stimulate CD28“ CD8+ T cells with PMA to demonstrate that these cells produce TNF and IFNg. They conclude that these cells can be a source of TNF and IFNg, indeed upregulated in the bone marrow. It is not surprising that CD28“ CD8+ T cells produce IFNg and TNF, as any T cell other than naive would. Moreover, additional cells types including CD4 and macrophages can be source of these cytokines at different levels.

5. As for APRIL, TNF is investigated in some parts of the paper but it is not clear whether it plays a role in the biology of memory cells. Any data on TNF expression in the BM of SOD1 ko mice? Actually, the correlation of TNF with age seems stroger than that of IFNg in Figure 4b.

Minor points

1. TNF, not TNFa is the correct nomenclature
2. Figure 5 D-G: y axes should start at 0
3. Figure 5 F-G: statistics graphics covers the IFNg+ vitC- culture condition

<u>First Revision – authors' response</u>

<u>21-Oct-2016</u>

Reviewer 1
Comments to the Author

European Journal of
Immunology

In the present manuscript, Pangrazzi et al. analysed the impact of aging in the long-term maintenance of effector/memory T cells and plasma cells in the bone marrow (BM). In particular, they analyzed the production of survival factors (IL-7 and APRIL) as well as pro-inflammatory factors (IL-15 and IL-6) in BM mononuclear cells (BMMCs) from individuals of different ages. During aging, they observed an increase of IL-15 and IL-6, which correlates with the expansion of harmful CD8+ CD28+ senescent cells, and a decrease of IL-7 and APRIL, which are important survival factors for the maintenance of long-term effector/memory CD4+ T cells and plasma cells, respectively. They also correlated the production of IL-15 and IL-6 with IFN-γ and ROS levels, thus suggesting that anti-oxidants may improve immunological memory during aging. The results are interesting but key controls are missed and some experiments need a more extensive description.
Major points:
1) Figure 2. In this figure the authors evaluated the production of IL-15, APRIL, IL-6 and CXCL12 by intracellular staining of BMMCs. BMMCs are an heterogeneous population, thus measuring the level of cytokine production by intracellular staining is not informative, especially because in the Figure 3 they performed a deeper analysis of the BM cell types producing each cytokine. Instead, it is pivotal to demonstrate that all cytokines are secreted. Thus, the quantitative measurement of each cytokine in the culture supernatant of BMMCs from people of different ages is necessary.
Following the suggestions of the reviewer, the levels of APRIL, IL-6 and CXCL-12 have now been measured by ELISA in BMMC supernatants from 9 additional persons of different ages confirming the results obtained by qPCR and intracellular staining. As pointed out in the 2

introduction, IL-15 is mostly transpresented by IL-15-transpresenting cells in order to be biologically active. The cytokine is secreted at a very low concentration. Furthermore, the affinity of the secreted form of IL-15 for the IL-15Rβ is much lower compared to the transpresented form. Despite this, we tried to measure the expression of IL-15 in the supernatants but the concentrations were obviously under the detection level of the assay. The methods and the ELISA measurements are now mentioned in the text ("Results" page 5 and "Materials and Methods" page 17). They are summarized in Figures 2 E-G and mentioned in the Legend to Figure 2.
2) Figure 3 and Supp. Fig.1: In figure 3, the authors show the intracellular staining of cytokine production performed in CD11chi, CD14+ and CD34+ BMMCs. An example of the gating strategies used to identify the three population is also shown in Supp. Fig.1. However, no data on the percentage of each population in all analysed individuals are shown.
The percentage of each population in all individuals is now shown in the new Suppl. Figure 1 B-D .
Moreover, the dot plots of the intracellular staining of all cytokines analysed in each population from at least a representative young and old individual should be shown in Supp Fig. 1.
The dot plots of the intracellular staining of all cytokines in each cell population in one representative young and one old sample are shown in a new Suppl. Figure 2 and are described in the respective figure Legend.
3) Figure 4 C and D. In panel C, the authors show the percentage of IFN-γ and TNF-α production in the CD8+ CD28- T cell population from the BMMCs of N=12 individuals. Several details are

European Journal of Immunology

missed. Which is the age of the 12 individuals? How do they isolate CD8+ CD28- T cells for intracellular staining? Similar details are missed in the panel E that show ROS production in PBMCs and BMMCs from the same donors. Which donors? Are aged donors or young donors?
The original Figure 4 C has been removed from the manuscript and replaced by a new Figure 4, containing Figures 4 E, F and G. Figure E shows a correlation between the percentage of CD8+CD28- T cells within CD8+ T cells in the BM in BMMCs from 20 persons. In Figures 4 G and F two age group with 10 persons in each group were analyzed (see point 4). The new data are also mentioned in the text of the revised version (page 7). The age of the probands

in the groups are now mentioned in the Figure legend when not shown in the graphs. CD8+CD28- T cells, as well as CD8+CD28+ T cells and CD4+ T cells are gated inside the BMMC population following immunofluorescence staining. CD8+CD28- T cells were not isolated for analysis.

4) Moreover, it is pivotal to compare: 1. The % CD8+ CD28- T cells producing IFN-γ and TNF-α between younger and older individuals; 2. The % of IFN-γ and TNF-α produced by CD8+ CD28-, CD8+ CD28+ and CD4+ T helper cells in both young and old individuals.
As suggested by the reviewer and already mentioned in point 3, we now show a correlation between the percentage of CD8+ CD28- T cells and age, as well as the percentage of IFN-γ- and TNF- producing cells in CD8+CD28+, CD8+CD28- and CD4+ T cell subpopulations in two age groups (<60 years, mean 54±4.8, range 46-59 and > 65 years, mean 72±4.7, range 66-79) in Figures 4 F and 4 G of the revised version. Details about the experiments are described in the Figure legend and on page 7 of the "Results" section of the revised version.

5) Figure 5. The authors correlate ROS production with cytokine expression in BMMCs (Panel A-C). Again, the details on the experimental procedure and population of BMMCs are missed. They reported the number of individuals (N=15) but not the range of age. In panels D to G, no details on the age of the individuals are reported.
Following the suggestion of the reviewer, details of the experimental procedure and the age of the persons analyzed are now reported in the legend to Figure 5.

6) Moreover, in these experiments the authors used PBMCs instead BMMCs, "in view of the increased sensitivity of PBMCs to cytokines compared to BMMCs". In Figure 3, IL-15 and IL-6 are highly produced by CD11chi cells from BMMCs, thus it is not comprehensible why they decided to perform these experiments in PBMCs, which, as shown in Figure 4E, produce very lower levels of ROS compared to BMMCs. These experiments and those reported in Supp Figure 2 should be performed in CD11chi cells from BMMCs.
A new Figure (Suppl.Figure 3) has been added to the revised version of the manuscript. Results from IFN-γ stimulation of IL-15 and IL-6 in CD11chi cells within BMMCs are shown. No induction of both cytokines could be observed in this population following IFN-γ stimulation. We believe that the BM proinflammatory environment induces higher IL-15, IL-

6 and ROS levels compared to PBMCs *in vivo*, which cannot be further increased by *in vitro* IFNγ stimulation. For this reason we considered PBMCs the more suitable model to study the stimulation of IL-15, IL-6 and ROS *in vitro*. This is now also mentioned in the "Results" section of the revised version (page 9).

7) Moreover, the authors correlate the accumulation of ROS with an accumulation of CD8+ CD28- T cells. However, no data on the percentage of CD8+ CD28- T cells in BMMCs from young and old individuals are reported. Finally, to correlate ROS accumulation with the survival of CD8+ CD28- T cells, these cells should be cultured in the presence or absence of NAC and Vit C and their survival rates should be evaluated.

We did not correlate ROS accumulation with the accumulation of CD8+CD28- T cells and did not study the survival of this cell population. This must have been a misunderstanding. We only suggest ("Discussion" section) that decreased levels of IL-15 and IL-6 promoted by ROS scavengers may have a negative impact on CD8+CD28- T cell survival, since a reduction in CD28- T cell apoptosis in the presence of IL-15 has been documented [34, 35]. Data on the percentage of CD8+CD28- T cells in persons of different age are shown in Figure 4 E (see also point 3).

Reviewer: 2

Comments to the Author

The paper by Pangrazzi et al. investigates the association between inflammation and ROS metabolism on the production of many factors that are important for the survival and differentiation of memory CD8+ T cells in the bone marrow. Most important, the authors analyze the impact of age. The manuscript reports some interesting observations, i.e. the association of age with the production of the proinflammatory molecules IL-15, IL-6 and IFN-5

g. This is not terribly surprising, as "inflammaging" is known to occur in the elderly, however, analysis are performed in human bone marrow and for this reason are of some interest. Unfortunately, the paper lacks a main clear focus, is descriptive in most of its parts and appears fragmented. Some data are reported but are not thouroughly investigated, therefore it is difficult to assess the impact of some powerful correlations (e.g. APRIL) with the main message of the manuscript, i.e. memory CD8+ T cell survival. Finally, it is difficult for the reviewer to understand whether the focus is on total bone marrow memory CD8+ T cells, or on CD28– terminally differentiated T cells, abundant in the elderly.

We agree with the reviewer that the paper appears descriptive in some parts. We believe that it was necessary to describe in detail the age-related changes in the expression of molecules involved in memory T cell and plasma cell maintenance in the BM, since this has not been reported so far. This work focuses mainly on effector/memory cell survival factors and investigates how proinflammatory molecules and ROS can regulate their expression in the BM. Experiments with T cells were only performed to study their role in the production of proinflammatory molecules, thus assessing their possible contribution to inflamm-aging. The focus of the paper is thus on memory cell survival factors and not on memory cells themselves.

Major concerns:

1. the manuscript relies mostly on correlations. When correlating age with the production of proinflammatory proteins, the authors use flow cytometry to define the production of IL-6 and IL-15 by BMMC. In this regard, the authors normalize the data vs. the isotype control for every given sample. I have two major objections to this approach:

a. in the reviewer's experience measuring IL-15 by FACS in humans is tricky. Additional raw data should be provided to assess the validity of the assay. The authors mentioned they validated the

antibody staining by pre-incubating the primary antibody with recombinant IL-15 or IL-6. While this demonstrates the specificity of the assay, it does not take into account 6

the possibility that the staining with the primary antibody increases background fluorescence. Such a possibility can be assessed by carefully titrating the antibody on cell lines that express or not the antigen (not mentioned in Methods).
Those cells not expressing the antigen (crossvalidated by qPCR for example or by western blot) should not give any staining. Staining with the isotype control does not give useful information on the unspecific binding of the antibody but only gives information on the possibility that the primary antibody binds Fc receptors.
b. As a consequence to point 1a, normalizing data vs. the isotype control is incorrect. Authors should also consider that it is difficult to titrate isotypes and thus determine whether non-specific binding other than that to Fc receptors is occurring.
We appreciate the reviewer's comments and agree that measuring IL-15 by FACS is indeed tricky. Following the reviewer's suggestion, we now show our FACS data without normalization against isotype controls. Since MFIs of the isotype controls were around 0 in most of the cases, the new data are not much different from the ones previously shown. To exclude the possibility that the primary Ab increases background fluorescence, we again followed the reviewer's advice and measured IL-15 expression in cells producing high amounts of this cytokine (SGBS cell line) and in cells not producing it (Jurkat cells). The results of these control experiments are now shown in Suppl. Figure 5 and described in the Figure legend as well as in the "Materials and Methods" section, in a new paragraph "Ab specificity tests", (page 16 of the revised version). It is clear that the IL-15 antibody is specific against the antigen since only cells expressing IL-15, crossvalidated by qPCR, show a staining. In IL-15-expressing cells, the IL-15 upregulation after stimulation with IFN-γ measured by FACS reflected the situation described at the mRNA level (Suppl Figure 5 C and 5 D and respective figure legend).
2. Could western blot of proinflammatory mediators be performed from BMMC lysates to confirm qPCR data?
To confirm qPCR data, we measured IFN-γ and TNF expression by ELISA in BMMC supernatants. This method additionally allowed us to measure the secretion of the two proinflammatory cytokines (see also our reply to Reviewer 1's comments point 1). The 7

results are shown in Figure 4 C-D and described under "Results" in the revised manuscript on page 7.
3. In figure 3, APRIL MFI is inversely correlated with age only when measured on CD34+ cells. However, CD34+ cells, despite enriched, are a minor population in the BM. The same correlation is seen on total BMMC presented in Figure 1. Is it therefore important to show data on total BMMC if it is known that CD34+ cells drive the correlation?
It is perhaps not absolutely necessary, but we believe that showing total BMMC is clearer and more informative. We therefore decided not to change this part of the paper.
Moreover, evaluating the presence of a given antigen by FACS on total BMMC is difficult because BM harbours many different cell types with different autofluorescence. If those cells with the highest autofluorescence are the most abundant, the same cells will "hide" the

positivity of other cells and thus decrease sensitivity. This can be prevented by dedicated gating strategies. Unfortunately, not enough information is provided in this regard.
In order to determine whether CD34+ cells have high autofluorescence, we checked the position of this cell population in the scatter plot. After gating out dead cells, we gated the CD34+ subpopulation in BMMCs and displayed it in the scatter plot. As shown in the new Suppl.Figure 6 of the revised version, CD34+ cells are typically located in a small area in the plot and they have low granularity. This suggests that CD34+ cells have low autofluorescence. These results are described in the legend of Suppl.Figure 6 and in the "Materials and Methods" section, in the paragraph "Abspecificity tests", page 16 of the revised manuscript.
4. Figure 4c: authors stimulate CD28– CD8+ T cells with PMA to demonstrate that these cells produce TNF and IFNg. They conclude that these cells can be a source of TNF and IFNg, indeed upregulated in the bone marrow. It is not surprising that CD28– CD8+ T cells produce IFNg and TNF, as any T cell other than Naïve would. Moreover, additional cells types including CD4 and macrophages can be source of these cytokines at different levels.
It was not our intention to claim that only CD8+CD28- T cells produce IFN-γ and TNF. Of course, other cell types can also produce these cytokines. We apologize for this misunderstanding. Our goal was to demonstrate that the high production of IFNγ and TNF by CD8+CD28- T cells may be important for the development of an inflammatory 8

environment in the BM in old age. As suggested by reviewer 1 (point 4), we now show the expression of IFN-γ and TNF after PMA/IONO stimulation in CD8+CD28+, CD8+CD28- and CD4+ T cell subpopulations in younger and old donors. We demonstrate that the production of proinflammatory cytokines in CD8+ CD28- T cells is much higher compared to CD8+CD28+ and CD4+ T cells. Results are shown in the new Figures 4 F and 4 G and described on page 7 of the revised manuscript.
5. As for APRIL, TNF is investigated in some parts of the paper but it is not clear whether it plays a role in the biology of memory cells. Any data on TNF expression in the BM of SOD1 ko mice? Actually, the correlation of TNF with age seems stroger than that of IFNg in Figure 4b.
As rightly assumed by the reviewer, the correlation of TNF with age is stronger than the one of IFN-γ. We now show the data on TNF expression in the BM of SOD1-/- mice in the Figures 6 F-H and we describe them in the figure legend and in the text on page 10 of the revised manuscript.
Minor points
1. TNF, not TNFa is the correct nomenclature
2. Figure 5 D-G: y axes should start at 0
3. Figure 5 F-G: statistics graphics covers the IFNg+ vitC- culture condition
The mistakes listed under "minor points" have been corrected.

Second Editorial Decision

16-Nov-2016

Dear Prof. Grubeck-Loebenstein,

It is a pleasure to provisionally accept your manuscript entitled "Inflamm-aging• influences immune cell survival factors in human bone marrow" for publication in the European Journal of Immunology. For final acceptance, please follow the instructions below and return the requested items as soon as possible as we cannot process your manuscript further until all items listed below are dealt with.

Please note that EJI articles are now published online a few days after final acceptance (see Accepted Articles: http://onlinelibrary.wiley.com/journal/10.1002/(ISSN)1521-4141/accepted). The files used for the Accepted Articles are the final files and information supplied by you in Manuscript Central. You should therefore check that all the information (including author names) is correct as changes will NOT be permitted until the proofs stage.

We look forward to hearing from you and thank you for submitting your manuscript to the European Journal of Immunology.

Yours sincerely,
Nadja Bakocevic

on behalf of
Prof. Silvano Sozzani

Dr. Nadja Bakocevic
Editorial Office
European Journal of Immunology
e-mail: ejied@wiley.com
www.eji-journal.eu
